# Microbubble-Assisted Cleaning-in-Place Process for Ultrafiltration System and Its Environmental Performance

**DOI:** 10.3390/membranes13040424

**Published:** 2023-04-10

**Authors:** Monique Mi Song Chung, April J. Arbour, Jen-Yi Huang

**Affiliations:** 1 Department of Food Science, Purdue University, West Lafayette, IN 47907, USA; 2 Environmental and Ecological Engineering, Purdue University, West Lafayette, IN 47907, USA

**Keywords:** milk concentration, dairy processing, membrane fouling, permeate flux, fine bubbles, life cycle assessment, environmental impact, sustainability

## Abstract

Membrane filtration is a key technology in dairy processing for the separation of dairy liquids to clarify, concentrate, and fractionate a variety of dairy products. Ultrafiltration (UF) is widely applied for whey separation, protein concentration and standardization, and lactose-free milk production, though its performance can be hindered by membrane fouling. As an automated cleaning process commonly used in the food and beverage industries, cleaning in place (CIP) uses large amounts of water, chemicals, and energy, resulting in significant environmental impacts. This study introduced micron-scale air-filled bubbles (microbubbles; MBs) with mean diameters smaller than 5 μm into cleaning liquids to clean a pilot-scale UF system. During the UF of model milk for concentration, cake formation was identified as the dominant membrane fouling mechanism. The MB-assisted CIP process was conducted at two bubble number densities (2021 and 10,569 bubbles per mL of cleaning liquid) and two flow rates (130 and 190 L/min). For all the cleaning conditions tested, MB addition largely increased the membrane flux recovery by 31–72%; however, the effects of bubble density and flow rate were insignificant. Alkaline wash was found to be the main step in removing proteinaceous foulant from the UF membrane, though MBs did not show a significant effect on the removal due to the operational uncertainty of the pilot-scale system. The environmental benefits of MB incorporation were quantified by a comparative life cycle assessment and the results indicated that MB-assisted CIP had up to 37% lower environmental impact than control CIP. This is the first study incorporating MBs into a full CIP cycle at the pilot scale and proving their effectiveness in enhancing membrane cleaning. This novel CIP process can help reduce water and energy use in dairy processing and improve the environmental sustainability of the dairy industry.

## 1. Introduction

Milk is a complete food that contains high-quality protein and is an excellent source of calcium and lactose. However, raw milk can carry pathogenic bacteria, including *Brucella*, *Campylobacter*, *Cryptosporidium*, *E. coli*, *Listeria*, and *Salmonella*, and can spread diseases such as tuberculosis and typhus [1]. Since the 19th century, milk has been traditionally processed by heat treatment, which is the common practice to inhibit microbial growth in milk. However, some changes in milk’s nutritional and sensory characteristics may unavoidably occur following heat treatment that are adverse to consumer acceptance [2].

Membrane technology has revolutionized the dairy industry since the 1970s [1]. Different types of membranes are used in the industry for various purposes. Microfiltration as a pretreatment can remove bacteria from milk without exposure to heat treatment, defat whey for producing whey protein concentrate, and fractionate milk proteins [3]. Ultrafiltration (UF) can be applied for whey separation and conversion into refined proteins, milk protein concentration, and standardization during cheese making, and lactose-free milk production [4,5,6]. Nanofiltration and reverse osmosis can be used to demineralize and dehydrate whey, respectively [3,7]. However, the bottleneck of membrane technology is membrane fouling, i.e., accumulation of unwanted deposits on the membrane surface or inside membrane pores, which leads to a reduction in permeate flux and consequently losses of separation efficiency and process productivity over time [5,8,9]. Therefore, regular membrane cleaning is required to minimize these phenomena.

Membrane cleaning is an essential step to maintain membrane performance, increase membrane life span, and reduce the costs of membrane replacement [4,5]. Cleaning in modern food manufacturing sectors is often performed by cleaning-in-place (CIP) systems [8,10]. Conventional CIP operations are composed of five steps: water prerinse, alkaline wash, intermediate water rinse, acid wash, and final water rinse [11]. Water rinsing steps are generally employed to remove excess product residue or detergents from the production line; an alkaline wash enables the removal of organic residues such as fats and proteins; and an acid wash is responsible for removing mineral scales and neutralizing residual alkaline cleaner [12,13]. To comply with stringent food safety and hygiene standards, CIP operations require the use of large amounts of water, chemical cleaning agents, and energy, resulting in significant environmental impacts [14].

Recently, small air bubbles with diameters less than 50 µm, also known as microbubbles (MBs), have distinct properties from millimeter-sized bubbles, including long residence time in liquid, large surface-to-volume ratio, high gas dissolution rate, and generation of high energy and free radicals when they burst [15,16]. MBs have been applied in various food-related areas, such as increasing soil removal from baby spinach during washing [17], promoting inactivation of *Escherichia coli* and *Salmonella Typhimurium* on leafy vegetables [18], enhancing salt diffusion into pork meat during brining [19], and intensifying yeast production [20]. Due to the hydrophobic surfaces, MBs have also proven effective in the cleaning of organic deposits and pollutants, including milk deposit removal from heat transfer surfaces [21], biofilm detachment from the membrane surface [22], food oil deposit removal from the microfiltration membrane [23], fouling reduction on the reverse osmosis membrane [24], and biodiesel removal from sand [25].

The objective of this study was to evaluate the effect of MBs on the performance of the CIP operation for a pilot-scale UF system used for milk concentration. Operating parameters studied included the MB number density and flow rate of the cleaning liquid. Moreover, a comparative life cycle assessment (LCA) was performed to quantify the environmental benefits of incorporation of MBs into CIP operation. The results showed that MB-incorporation significantly improved the cleaning performance, in terms of membrane flux recovery, by up to 72%, which led to an up to 37% reduction in the environmental impacts associated with CIP operation.

## 2. Materials and Methods

### 2.1. UF of Whole Milk

**Figure 1:**
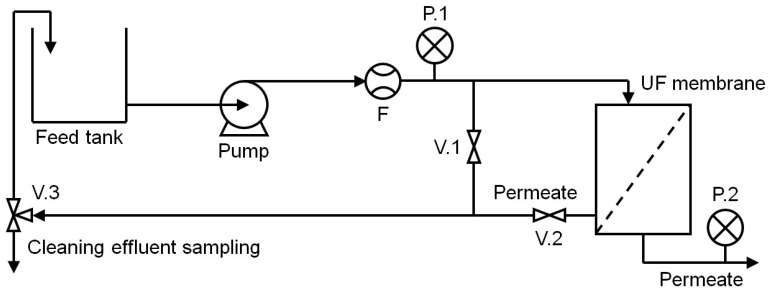
Schematic of pilot-scale UF system.

Milk concentration was performed using a pilot-scale UF system, as shown in Figure 1. The system consisted of a feed tank, a three-phase pump motor (5 HP, 3450 rpm; Baldor ABB, Fort Smith, AR, USA), a spiral polyethersulfone UF membrane (GR73PE-3838/80, Alfa Laval, Richmond, VA, USA) with a molecular weight cutoff of 10 kDa and total active membrane area of 3.4 m^2^, two restriction valves (V.1, V.2), a three-way valve (V.3), two pressure gauges (P.1, P.2), a flowmeter (F), and a permeate tank. Fifty gallons of model milk were prepared by reconstituting whole milk powder (Great American Spice Company, Rockford, MI, USA) to a concentration of 0.5% *w/v* in RO water. The concentration process was conducted by pumping the model milk through the UF system at a flow rate of 130 L/min for 40 min, and the transmembrane pressure (TMP) was controlled at 30 psi. The membrane was conditioned for 30 min with RO water at a flow rate of 130 L/min and TMP of 20 psi before each concentration experiment.

### 2.2. MB Liquid Generation and Characterization

MBs were infused into cleaning liquids using a Nikuni regenerative turbine pump KTM20 (KTM65S; Nikuni Co., Kawasaki, Japan) where air bubbles are generated via breakup by flow turbulence and vortices. The number of bubbles in the liquid was controlled by air injection, and two air flow rates (5 and 10 L/min) were tested. The number density and mean size of MBs in water were determined using a particle counter (PC3400, Chemtrac, Norcross, GA, USA). MB-infused alkaline and acid solutions were not characterized in this study due to the limitation of the particle counter which cannot be operated with alkaline and acid solutions, according to the manufacturer.

### 2.3. Membrane Cleaning Experiments

A 7-step CIP process was performed to clean the UF membrane fouled during model milk concentration. Table 1 shows all the steps and their operating temperatures and periods. To avoid the shrinkage of membrane pores at low pH [26], additional alkaline wash and water rinse steps were conducted after the acid wash step to recondition the membrane into an alkaline pH. All the steps were conducted at constant TMP (20 psi) and flow rate of the cleaning liquid, and two different flow rates (130 and 190 L/min) were tested in this study. To evaluate the effect of MBs on the performance of the CIP process, MBs were incorporated into the cleaning liquids of the water rinse, alkaline wash, and acid wash steps. The CIP process, operated with MB-free cleaning liquids, was conducted as the control.

After the full CIP process, the cleanliness of the membrane was determined by its water permeate flux, measured at a TMP of 20 psi and a flow rate of 130 L/min over 10 min. The cleaning performance was evaluated based on the recovery of membrane flux, namely the ratio of water permeate flux of the cleaned membrane (${\dot{J}}_{cleaned}$) to that of the fresh membrane (preconditioned with water only; ${\dot{J}}_{fresh}$), as shown in Equation (1) [27].

(1) $$Membrane\;flux\;recovery=\frac{{\dot{J}}_{cleaned}}{{\dot{J}}_{fresh}}$$

Repeated concentration-cleaning testing was performed using the same membrane throughout this study. Therefore, to minimize the foulant left on the membrane after every cleaning test, the same CIP process was conducted again but with increased time, temperature, and/or chemical concentration, comprising prerinse (20 °C, 15 min), alkaline wash (0.1% NaOH, 55 °C, 20 min), intermediate rinse (20 °C, 15 min), acid wash (0.2% HNO_3_ + 0.2% H_3_PO_4_, 55 °C, 20 min), alkaline reconditioning (0.1% NaOH, 43 °C, 5 min), and final rinse (20 °C, 15 min). Between the intermediate rinse and acid wash steps, an additional enzymatic cleaning step using 0.5% Filzym 131 (Realco, Louvain-la-Neuve, Belgium) at 43 °C for 45 min) followed by a water rinse step (20 °C, 15 min) were applied. Furthermore, a disinfecting step using a 0.05% peroxyacetic-based sanitizer (Oxywave, Madison Chemical Co., Madison, IN, USA) at 32 °C for 20 min was applied after every three cleaning tests, as recommended by the membrane manufacturer.

Membrane fouling by the milk model solution was characterized using the modified Hermia’s model (Equations (2) and (3); [28]).

(2) $$\frac{dJ}{dt}=-k\cdot {\left(J-J^{*}\right)\cdot J}^{2-n}$$

(3) $$J=\frac{dv}{dt}\times \frac{1}{A}$$

where *J* is the permeate flux, *t* is the filtration time, *k* is the resistant coefficient, J* is the permeate flux at steady state, *n* is the blocking index, *v* is the permeate volume, and *A* is the effective membrane area. Specifically, the dominant fouling mechanism was determined by the linearity of the flux curves of *t*/*v* − *v*, *t*/*v* − *t*, and Ln(*t*) − *v*, which correspond to cake formation, standard blocking, and intermediate blocking mechanisms, respectively [29].

### 2.4. Determination of Protein and Fat Removal

Membrane fouling during the UF of milk is mostly due to the precipitation of microorganisms, proteins, fats, and minerals [30]. To better evaluate the cleaning performance of each step of the CIP process, the cleaning liquid (i.e., UF retentate) was sampled after each cleaning step for measurements of protein and fat concentrations to determine the amounts of proteins and fats removed. The protein concentration was quantified by the micro BCA (MBCA) protein assay [31,32]. One milliliter of the sample was mixed with 1 mL of prepared MBCA reagent (reagent A:reagent B:reagent C = 25:24:1) and incubated at 60 °C for 60 min. The absorbance of the mixture at a wavelength of 562 nm was determined using a spectrophotometer. The protein content of the sample was calculated using the standard curve built with bovine serum albumin [33]. The fat concentration was determined using the Mojonnier method [34] performed by Dairy One (Ithaca, NY, USA).

### 2.5. Statistical Analysis

All experiments were performed in triplicate. Data are expressed as mean ± standard deviation and were statistically analyzed by the one-way analysis of variance (ANOVA) followed by Tukey’s test to compare the means of different test groups, with a significance level of 0.05. All statistical analyses were performed using the SAS 9.4 software (SAS Institute, Cary, NC, USA).

### 2.6. Life Cycle Assessment

In addition to production-based metrics [35], the efficiency of a cleaning process can be measured by environmental footprint indicators [14,36]. This study used life cycle assessment (LCA) approaches to analyze the environmental consequences of the application of an alternative CIP process in a simplified concentrated milk manufacturing line. LCA is a standardized method widely used for quantifying the environmental impacts associated with food processes and products [37].

#### 2.6.1. Goal and Scope

The goal of this LCA study is to compare the environmental performance of a new CIP method, based on the incorporation of MBs, to that of a conventional CIP process for a pilot-scale UF system employed for whole milk concentration. The results are intended to provide food manufacturers and cleaning system designers and operators with quantitative indicators of the environmental benefits of the new MB-assisted cleaning technology.

The simplified concentrated milk production was analyzed from gate to gate, including the concentration and CIP processes. The upstream stages (feed production, cow farming and milking, and transportation) and milk packaging were not considered. The functional unit (FU) of this comparative LCA was defined as one production cycle that included a 40-min concentration process operated under the UF condition described in Section 2.1 and a 7-step CIP process (Table 1; with or without MBs incorporated) to recover the membrane flux to a preset level (membrane flux recovery = 0.32, determined based on the result of control (MB-free) CIP process). The operating condition of the CIP process that resulted in the largest flux recovery enhancement by MB incorporation (flow rate = 130 L/min, MB number density = 10,569 bubbles/mL; reported in Table 2) was selected for assessment.

#### 2.6.2. Life Cycle Inventory

The life cycle inventory (LCI) included the resources required for one concentrated milk production cycle, such as milk powder, water, and chemicals, as well as the utilities for pumping and heating. The foreground inventory data were collected from pilot tests, and all the background data including electricity, natural gas, and productions of milk powder and chemicals were adapted from the ecoinvent database v3.0 [38]. Processing equipment was not included since it is considered a long-term infrastructure.

#### 2.6.3. Life Cycle Impact Assessment

The environmental impacts of one production cycle were determined using the ReCiPe 2016 Midpoint (E) v1.02 method. This method characterizes eighteen midpoint indicators, including global warming, stratospheric ozone depletion, ionizing radiation, ozone formation, human health, fine particulate matter formation, ozone formation, terrestrial ecosystems, terrestrial acidification, freshwater eutrophication, marine eutrophication, terrestrial ecotoxicity, freshwater ecotoxicity, marine ecotoxicity, human carcinogenic toxicity, human noncarcinogenic toxicity, land use, mineral resource scarcity, fossil resource scarcity, and water consumption. All calculations were conducted using the SimaPro software v8.0.

## 3. Results and Discussion

### 3.1. Milk Fouling on UF Membrane

**Figure 2:**
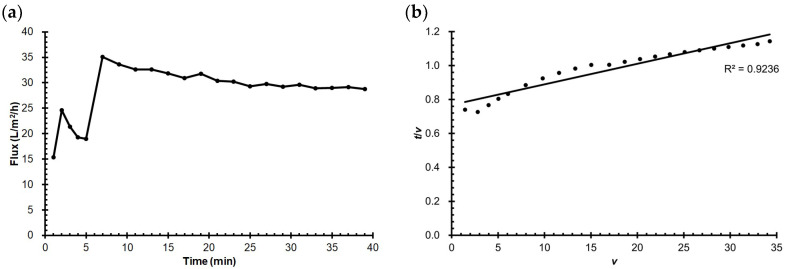
(**a**) Evolution of membrane flux during the concentration of model milk; (**b**) Fitting of permeate volume (*v*; L) and filtration time (*t*; min) during the UF of model milk to Hermia’s model.

During the 40-min concentration of model milk, the membrane flux reached 35.1 L/m^2^/h after the first 5 min for initial conditioning then gradually decreased to 28.8 L/m^2^/h over the rest of the process, as shown in Figure 2a, implying the formation of membrane fouling by milk. The goodness of fit of Hermia’s model to the data on permeate volume (*v*) and filtration time (*t*) was measured by the coefficient of determination (*R*^2^). Cake formation (Figure 2b) showing the highest *R*^2^ value (0.92) was identified as the dominant fouling mechanism of milk concentration in this study, followed by standard blocking (*R*^2^ = 0.90) and intermediate blocking (*R*^2^ = 0.87). Cake formation is a reversible type of fouling with a layer of foulant loosely attaching to the membrane surface that can be effectively removed by physical cleaning [39]. Ref. [40] investigated the mechanisms of membrane fouling formed during the microfiltration of skim milk and found foulants on the membrane surface and inside the membrane pores. In the shear-enhanced UF of whole milk, complete pore blocking, cake formation, and intermediate pore blocking were identified as the main fouling mechanisms [41].

### 3.2. MB Characterization

The MBs generated in water showed a unimodal narrow size distribution. The flow rate of air injection into the Nikuni pump had an insignificant effect on the mean MB diameter, of 4.51 μm for 5 L/min and 4.26 µm for 10 L/min. However, increasing the air flow rate was found to markedly decrease the MB number density, from 10,569 bubbles/mL for 5 L/min to 2021 bubbles/mL for 10 L/min. This could be due to a larger volume of air infused into the water tends to induce the formation of more millimeter-sized bubbles that can rise quickly and burst at the water’s surface.

### 3.3. Membrane Cleaning

Table 2 compares the cleaning performance, in terms of membrane flux recovery, of the control and MB-assisted CIP processes operated at different conditions. For the two flow rates tested, the incorporation of MBs significantly improved the performance of the CIP process. At the low flow rate (130 L/min), MB-assisted CIP showed 59–72% higher flux recovery than the control; however, increasing the density of MBs from 2021 to 10,569 bubbles/mL did not have a significant effect. Although increasing the flow rate to 190 L/min increased the flux recovery regardless of MB incorporation due to the higher shear exerted by the liquid flow on the foulant, MBs (10,569 bubbles/mL) had a less enhancing effect on cleaning, which increased the flux recovery by 31% compared to the control.

MBs have been reported to aid deposit removal by increasing momentum transfer due to bubble scrubbing [42] as well as energy released and shear flow generated by bubble collapse [24]. Moreover, in our previous study on MB-assisted cleaning of fouled heat transfer surface [21], MBs were observed to attach to milk foulant, resulting in enhanced removal. However, the spacer mesh placed to separate the two adjacent membranes and create the feed channel could limit the number of intact bubbles flowing across the spiral membrane, which can explain the insignificant improvement in flux recovery when increasing the bubble density in cleaning liquids. Further, when the flow rate increases, the resulting turbulence could increase the contact frequency between bubbles and the spacer mesh, making bubbles more susceptible to breakage. Therefore, MB-assisted CIP was found to have a smaller flux recovery enhancement over the control at the flow rate of 190 L/min than 130 L/min. The increased turbulence at a high flow rate could also shorten the time for MBs to attach to foulant and adsorb foulant molecules, leading to less cleaning enhancement. A similar trend was found in our previous study on MB-assisted cleaning of the microfiltration membrane fouled by food oily wastewater [23]. To more clearly reveal the effects of bubble density and flow rate on foulant removal, and characterize the mechanisms of MB-assisted cleaning of spiral membrane, visualization of the MB–foulant interaction during cleaning using in situ real-time imaging techniques, such as magnetic resonance imaging, is highly needed.

### 3.4. Protein and Fat Removal

**Figure 3:**
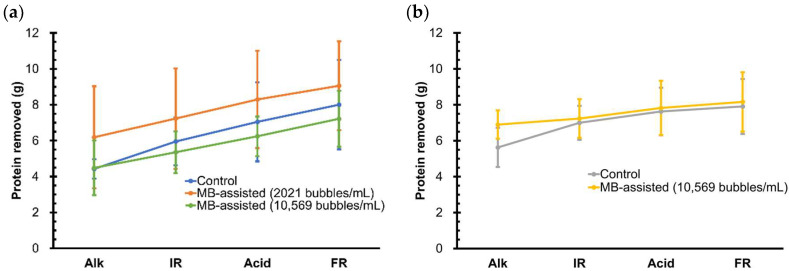
Effect of MB incorporation on the cumulative amount of protein removed over the CIP process operated at a flow rate of (**a**) 130 L/min and (**b**) 190 L/min. Alk: alkaline wash; IR: intermediate rinse, Acid: acid wash; FR: final rinse. All test groups were not significantly different from their respective control groups.

Figure 3 shows the cumulative amount of protein removed by each step of control and MB-assisted CIP processes. The prerinse step is not included here since it was mainly used to remove most of the remaining residue from the UF system after the milk concentration process, and the protein concentration of its effluent cannot be used to determine the amount of proteinaceous foulant removed. For all the cases, the amount of protein removed increased nearly linearly over the CIP process, in which alkaline was the most effective step as expected, corresponding to previous CIP studies [12,43,44]. Cleaning of the proteinaceous foulant involves an initial swelling stage, followed by uniform erosion, and a decay stage [45]. When the alkaline solution contacts the proteinaceous foulant, the foulant starts to swell and form voids within the protein matrix. Swollen foulant is more susceptible to removal by liquid shear force and diffusion compared to the unswollen foulant [46].

Due to the large variation in the protein concentrations of effluents collected from repeated experiments, the amounts of protein removed by the control and MB-assisted CIP processes showed no significant difference for all the cleaning conditions tested. This was primarily caused by the operational uncertainty of the pilot-scale UF system. Specifically, it is difficult to fully drain the cleaning liquid from the membrane housing after each cleaning step, hence the remaining residue is expected to affect the concentration of protein in the cleaning effluent of the following steps. Furthermore, for the MBCA protein assay used for protein concentration measurement, only 1 mL was sampled from the 50-gallon cleaning effluent for each analysis; therefore, a great spatial variation in the protein concentration across such a large volume of cleaning effluent can be expected. MBs have proved effective in removing proteins from microfiltration membrane by pyrolytic decomposition, which was characterized by temperature increases due to adiabatic compression when MBs collapsed [16]. Other studies also reported the protein removal capability of nanobubbles [15,47].

Fat has also been found to foul on the membrane during UF of whole milk [41]; however, in this study, fat was not detectable in the cleaning effluent collected from each cleaning step of the CIP process, regardless of MB incorporation and cleaning condition. This could be because the average milk fat globule (4 μm; [48]) is much larger than casein micelles (50–600 nm; [49]) and whey proteins (1.8–6 nm; [50]), and thus has a lower dissolution rate and also is more difficult to be sheared off during cleaning. Moreover, during MB-assisted cleaning, MBs (~4.4 μm) are expected to more likely to attach to and pick up molecules with a smaller size (proteins) than those with a similar size (fat globules).

### 3.5. LCA of MB-Assisted CIP Process

#### 3.5.1. Inventory Analysis

Table 3 summarizes the LCI data on one concentrated milk production cycle that included a control or MB-assisted CIP process. The electricity use for the CIP process was two to nearly three times as high as that for the concentration process due to the longer operation period (115 vs. 40 min). To fulfill the defined function unit of recovering membrane flux to the preset level of 0.32, the resources required for MB-assisted CIP were calculated based on the assumption that the membrane flux was recovered linearly during cleaning. Under the cleaning condition analyzed (flow rate = 130 L/min, MB number density = 10,569 bubbles/mL), MB-assisted CIP had a 72% higher flux recovery (Table 2), implying that it can reach the same level of cleanliness with 42% (obtained with $1/\left(1+0.72\right)$) less cleaning time (hence the uses of chemicals, water, and steam) than the control CIP. Therefore, although the Nikuni pump used to generate and circulate MB-infused cleaning liquids has a higher power than the pump used for the operation of the control CIP, its total electricity use for cleaning was lower, by 29%.

#### 3.5.2. Impact Assessment

**Figure 4:**
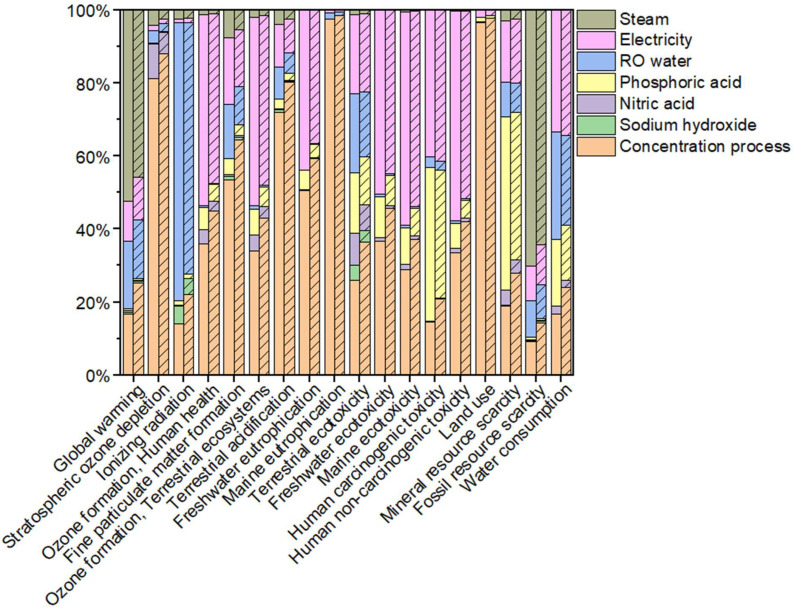
Contributions of concentration and CIP processes to total environmental impacts of simplified concentrated milk production. The CIP process is broken down into sodium hydroxide, nitric acid, phosphoric acid, water, electricity, and steam. Control (empty) and MB-assisted (patterned) CIP are compared.

Figure 4 shows the environmental profile of the simplified concentrated milk production including the control or MB-assisted CIP process. As the core stage of this comparative LCA, the eighteen midpoint environmental impacts of the CIP process were further broken down into the portions contributed by individual components used (i.e., chemicals, water electricity, and steam). For the control scenario, the CIP process predominated all the impacts (63–90%) with the exception of stratospheric ozone depletion (19%), fine particulate matter formation (47%), terrestrial acidification (28%), freshwater eutrophication (49%), marine eutrophication (3%), and land use (3%). Ref. [51] also reported that CIP was the largest contributor (>81%) to climate change, human toxicity, and marine ecotoxicity associated with the dairy pasteurization process. Moreover, CIP operations were found to be responsible for 70% of the water required for yogurt production [52] and 51% of the terrestrial ecotoxicity generated by egg yolk powder production [36]. In contrast, the LCA study of [53] on a milk fractionation process for the productions of cream, casein micelles concentrates, lactose, and whey proteins indicated that cleaning represented approximately 30% of the environmental impact.

Electricity use was considerably responsible for most of the impacts since coal is the dominant source of electricity generation (88%) in Indiana. Coal-fired thermal power plants release a number of toxins and pollutants into the air, rivers, streams, and lakes, including sulfur dioxide, nitrogen oxide, particulate matter, and mercury [54]. The steam used for heating cleaning liquids was generated by a natural gas steam boiler. Natural gas extraction and combustion directly resulted in fossil resource scarcity and global warming, respectively. Phosphoric acid use was the major contributor to mineral resource scarcity (47%) since phosphorus is not substitutable and phosphate reserves are limited [55]. Human carcinogenic toxicity was also mainly caused by phosphoric acid (42%). The concentration process accounted for significant portions of marine eutrophication (97%), land use (97%), stratospheric ozone depletion (81%), and terrestrial acidification (72%), primarily due to the milk powder used in this study. Raw milk is the most important input to milk-powder production (5.12–9.22 L for 1 kg milk powder; [56], emissions of nitrogen pollutants (ammonia and nitrate) from manure on dairy farms are the major sources of eutrophication and acidification of milk production [57]. Furthermore, [56] reported that emissions associated with wastewater treatment contributed approximately 40% to the marine eutrophication potential of milk powder processing. Ozone depletion can be attributed to the use of refrigerants for dairy farms’ milk cooler equipment, as well as emissions of methane from farm manure and nitrous oxide from fertilizers used for feed production in the milk production chain [57].

Table 4 compares the environmental impacts of the two simplified concentrated milk production scenarios. With a higher cleaning efficiency, the MB-assisted CIP process saved the cleaning time, and thus the inputs for one concentrated milk production cycle, resulting in lower total impacts for all of the eighteen indicators studied, by up to 37% (fossil-resource scarcity). Other environmental impacts that were markedly reduced by MB-assisted CIP included ionizing radiation (36%), global warming (34%), and mineral resource scarcity (32%). In contrast, the reductions in marine eutrophication and land use were negligible (~1%) since these two impacts were predominated by the concentration process. For the impacts particularly associated with the cleaning process, the MB-assisted CIP showed lower values than the control CIP by 30% (freshwater eutrophication) to 42% (ionizing radiation). Therefore, MB incorporation made the contribution of the CIP process to the total environmental impacts less dominant, with a 5–11% decrease, as shown in Figure 4.

## 4. Conclusions

The present study, for the first time, incorporated MBs into a full CIP cycle and investigated their effect on the cleaning of a pilot-scale UF system used for milk concentration under different bubble number densities and flow rates of cleaning liquids. The model milk tested was found to foul on the UF membrane mainly in the form of cake formation. Compared to the control CIP process, MB-assisted CIP showed a higher membrane flux recovery by up to 72%; however, increasing bubble density and flow rate did not show further improvement in this study. Despite the enhanced flux recovery, MBs had an insignificant effect on the amount of protein removed from the UF membrane and no fat removal was detected, which was mainly due to the operational uncertainty of the large-scale CIP process. The LCA on the simplified concentrated milk production revealed that the CIP process predominated most of the environmental impacts, which were mainly contributed by electricity use. Incorporation of MBs into the CIP process can reduce the impact by up to 37%. This study proved that MBs can be easily introduced into conventional CIP operations without major intervention in the existing processing equipment, which is a promising approach to increasing the efficiency of spiral membrane cleaning. Therefore, MB-assisted CIP has great potential for reducing the production costs and environmental footprint of filtered dairy products, including, not only high-protein milk but also whey protein isolate, casein, lactose, etc. Since the interaction between MBs and the milk foulant in the spiral membrane is still unclear, in situ real-time visualization is needed to provide further information about the cleaning mechanism of MB-assisted CIP. Moreover, characterizing the flow field over the spiral membrane with the presence of MBs by computational fluid simulations can help quantify the contact of MBs with foulant and the resulting forces exerted.

## Figures and Tables

**Table 1:** Operating conditions of the CIP process.

Step	Temperature (°C)	Time (min)
Water prerinse	20	15
Alkaline (0.1% *w*/*v* NaOH) wash	43	30
Intermediate water rinse	20	15
Acid (0.1% *w*/*v* HNO_3_ + 0.1% *w*/*v* H_3_PO_4_) wash	43	30
Second intermediate water rinse	20	5
Alkaline (0.1% *w*/*v* NaOH) pore reconditioning	43	5
Final water rinse	20	15

TMP was set at 20 psi for all the steps. Two flow rates (130 and 190 L/min) were tested for all the steps.

**Table 2:** Membrane flux recovery of control and MB-assisted CIP processes for UF membranes fouled by model milk.

**Flow Rate (L/min)**	130			190	
**CIP process**	Control ^⸸^	MB-assisted (2021 bubbles/mL)	MB-assisted (10,569 bubbles/mL) ^¶^	Control ^⸸^	MB-assisted (10,569 bubbles/mL) ^¶^
Flux recovery *	0.32 ± 0.06 ^bB^	0.50 ± 0.06 ^A^	0.54 ± 0.04 ^aA^	0.46 ± 0.03 ^aB^	0.61 ± 0.04 ^aA^

* Calculated by Equation (1). Values with different uppercase letters are significantly different at the same flow rate. ^⸸,¶^ Values of tests sharing the same superscript symbol which have different lowercase letters are significantly different.

**Table 3:** Inventory (for one production cycle) of simplified concentrated milk production.

Item (Unit)	Quantity	
**Concentration**		
Water (m^3^)	0.19	
Whole milk powder (kg)	1.10	
Electricity (kWh)	2.49	
**CIP**		
	*Control*	*MB-Assisted*
Water (m^3^)	1.62	0.94
Sodium Hydroxide (kg)	0.88	0.51
Nitric Acid (kg)	0.16	0.10
Phosphoric Acid (kg)	0.30	0.18
Steam (kg)	174	101
Electricity (kWh)	7.15	5.09

**Table 4:** Environmental impacts of simplified concentrated milk production including control or MB-assisted CIP process.

Impact Category (Unit)	Production with Control CIP	Production with MB-Assisted CIP
Global warming (kg CO_2_ eq)	2.04	1.36
Stratospheric ozone depletion (kg CFC11 eq)	7.09 × 10^−6^	6.54 × 10^−6^
Ionizing radiation (kBq Co-60 eq)	1.56 × 10^−1^	1.00 × 10^−1^
Ozone formation, human health (kg NO_x_ eq)	1.14 × 10^−3^	0.91 × 10^−3^
Fine particulate matter formation (kg PM2.5 eq)	2.00 × 10^−3^	1.66 × 10^−3^
Ozone formation, terrestrial ecosystems (kg NO_x_ eq)	1.28 × 10^−3^	1.02 × 10^−3^
Terrestrial acidification (kg SO_2_ eq)	1.06 × 10^−2^	0.95 × 10^−2^
Freshwater eutrophication (kg P eq)	2.56 × 10^−4^	2.18 × 10^−4^
Marine eutrophication (kg N eq)	9.77 × 10^−4^	9.67 × 10^−4^
Terrestrial ecotoxicity (kg 1,4-DCB)	2.36 × 10^−1^	1.69 × 10^−1^
Freshwater ecotoxicity (kg 1,4-DCB)	7.42 × 10^−3^	5.94 × 10^−3^
Marine ecotoxicity (kg 1,4-DCB)	70.42	54.87
Human carcinogenic toxicity (kg 1,4-DCB)	1.62	1.12
Human non-carcinogenic toxicity (kg 1,4-DCB)	58.43	46.53
Land use (m^2^a crop eq)	2.19 × 10^−1^	2.17 × 10^−1^
Mineral resource scarcity (kg Cu eq)	6.13 × 10^−4^	4.18 × 10^−4^
Fossil resource scarcity (kg oil eq)	5.78 × 10^−1^	5.78 × 10^−1^
Water consumption (m^3^)	1.61 × 10^−1^	1.12 × 10^−1^

## Data Availability

The data presented in this study are available on request from the corresponding author.
